# Supplementary figures and images for: Time-of-day changes in physician clinical decision making: A retrospective study
Source: PLoS One. 2021 Sep 17;16(9):e0257500. doi: 10.1371/journal.pone.0257500 (PMC8448311; doi:10.1371/journal.pone.0257500)

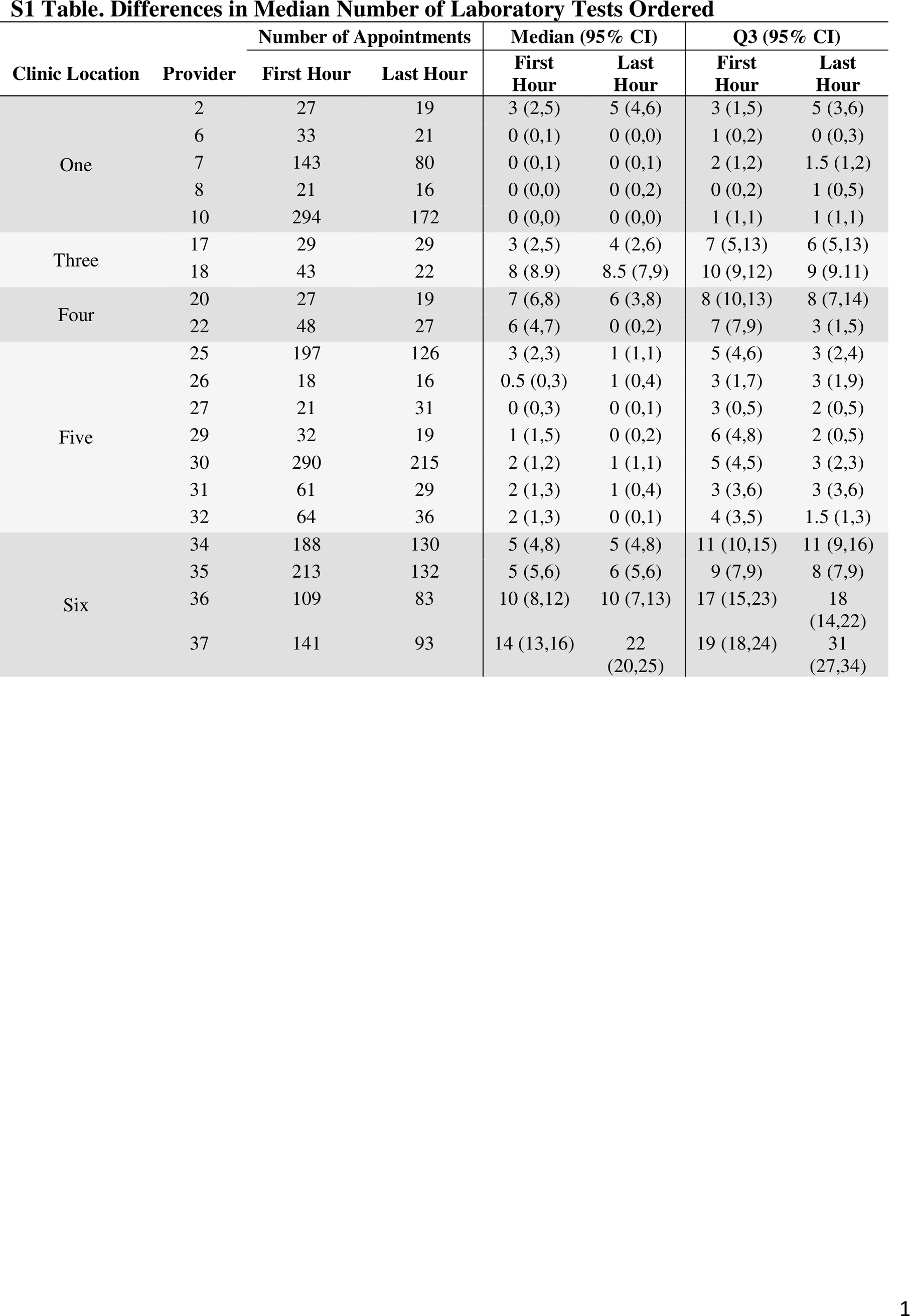

Supplement: S1 Table — (TIF) [file pone.0257500.s001.tif]

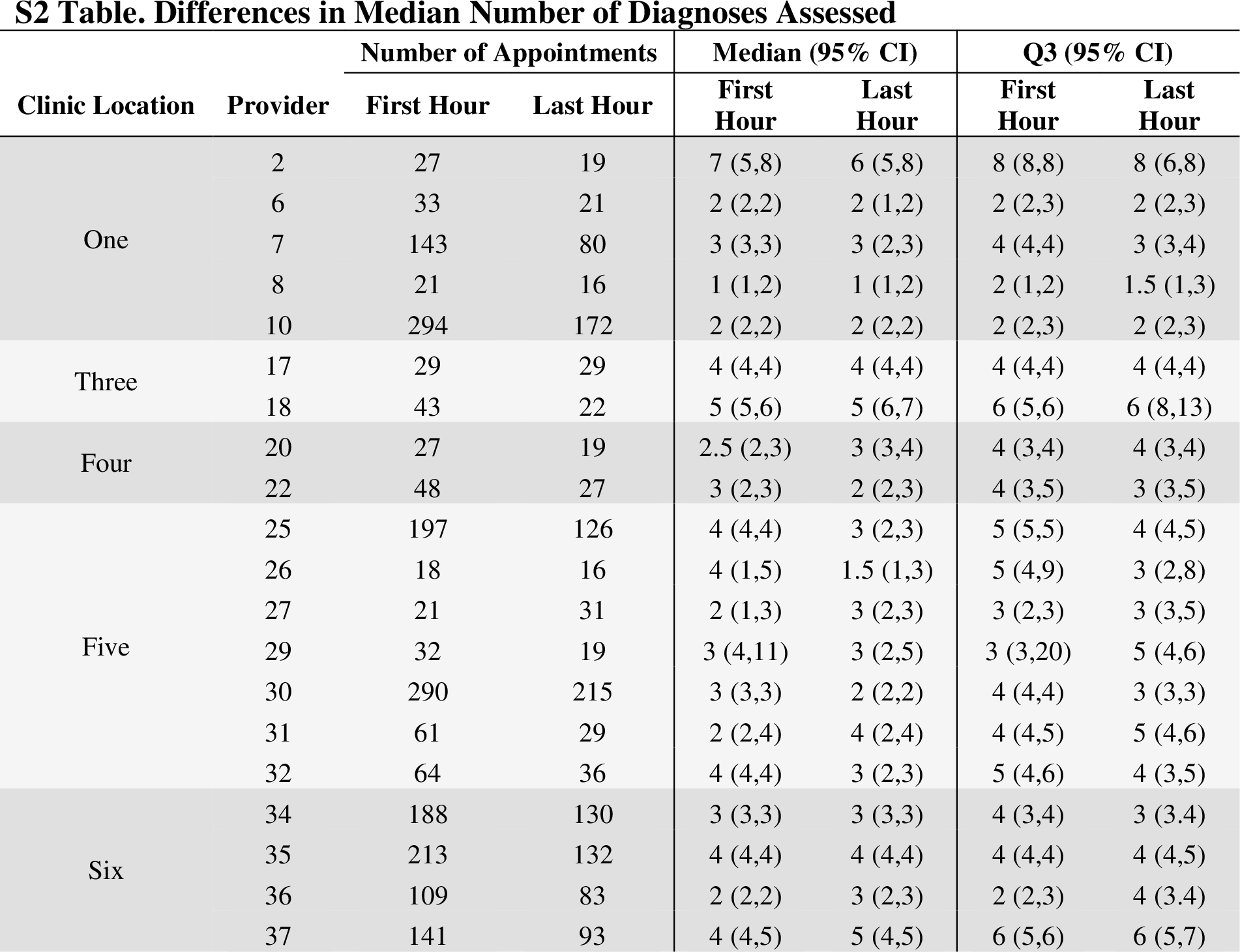

Supplement: S2 Table — (TIF) [file pone.0257500.s002.tif]
